# Mixed‐methods evaluation of a nurse‐led allergy clinic model in primary care: Feasibility trial

**DOI:** 10.1002/clt2.12180

**Published:** 2022-08-09

**Authors:** Vicky Hammersley, Margaret Kelman, Lynn Morrice, Marilyn Kendall, Mome Mukerjhee, Susan Harley, Jurgen Schwarze, Aziz Sheikh

**Affiliations:** ^1^ Usher Institute University of Edinburgh Edinburgh UK; ^2^ Health Data Research UK London UK; ^3^ Child Life and Health Centre for Inflammation Research University of Edinburgh Edinburgh UK

**Keywords:** allergy, primary care, quality of life

## Abstract

**Introduction:**

It is now widely acknowledged that there are serious shortcomings in allergy care provision for patients seen in primary care. We sought to assess the feasibility of delivering and evaluating a new nurse‐led allergy service in primary care, measured by recruitment, retention and estimates of the potential impact of the intervention on disease‐specific quality of life.

**Methods:**

Mixed‐methods evaluation of a nurse‐led primary care‐based allergy clinic in Edinburgh, UK undertaken during the period 2017–2021 with a focus on suspected food allergy and atopic eczema in young children, allergic rhinitis in children and young people, and suspected anaphylaxis in adults. Prior to March 2020, patients were seen face‐to‐face (Phase 1). Due to COVID‐19 pandemic restrictions, recruitment was halted between March–August 2020, and a remote clinic was restarted in September 2020 (Phase 2). Disease‐specific quality of life was measured at baseline and 6–12 weeks post intervention using validated instruments. Quantitative data were descriptively analysed. We undertook interviews with 16 carers/patients and nine healthcare professionals, which were thematically analysed.

**Results:**

During Phase 1, 426/506 (84%) referred patients met the eligibility criteria; 40/46 (87%) of Phase 2 referrals were eligible. Males and females were recruited in approximately equal numbers. The majority (83%) of referrals were for possible food allergy or anaphylaxis. Complete data were available for 338/426 (79%) patients seen in Phase 1 and 30/40 (75%) in Phase 2. Compared with baseline assessments, there were improvements in disease‐specific quality of life for most categories of patients. Patients/carers and healthcare professionals reported high levels of satisfaction, this being reinforced by the qualitative interviews in which convenience and speed of access to expert opinion, the quality of the consultation, and patient/care empowerment were particularly emphasised.

**Conclusion:**

This large feasibility trial has demonstrated that it is possible to recruit, deliver and retain individuals into a nurse‐led allergy clinic with both face‐to‐face and remote consultations. Our data indicate that the intervention was considered acceptable to patients/carers and healthcare professionals. The before‐after data of disease‐specific quality of life suggest that the intervention may prove effective, but this now needs to be confirmed through a formal randomised controlled trial.

**Trial Registration:**

ClinicalTrials.gov reference NCT03826953.

## 1 INTRODUCTION

A systematic review of worldwide delivery of allergy services^1^ highlighted what we have known from UK‐based studies for a decade, namely that primary care providers have limited training, expertise and confidence in allergy care and that demand for specialist allergy service far outweighs supply capabilities.^2^, ^3^, ^4^, ^5^ Primary and secondary care allergy pathways are inadequate, leading to poor referral processes, avoidable delays in management of allergic diseases and poor patient outcomes.^6^, ^7^ Specialist allergy care provision is patchy, but where it exists, evidence suggests that there are often unnecessary referrals to secondary care for conditions that could be dealt with in primary care settings.^8^

Lack of specialist allergy care provision has been highlighted in the UK for over 15 years.^9^, ^10^, ^11^ Jutel et al.^12^ suggested that there is a need to prioritise provision of allergy care within community settings rather than specialist settings, given the very large numbers of people now affected by allergic conditions.^4^ The 2013 Children's and Young People's Allergy Network Scotland (CYANS) report found that, across Scotland, primary care practitioners did not feel they have the skills or knowledge to provide good quality allergy care, especially around diagnostic testing for allergy, and the ability to interpret the results.^13^ A pilot study by Levy et al,^14^ run by a specialist allergy nurse and a GP with a special interest in respiratory disease and allergy, showed that a primary care intervention for allergy could effectively deal with the majority of cases of allergy seen in primary care, resulting in a reduction in inappropriate referrals into secondary care, an increase in self‐supported care for patients, and a saving in costs. These data were however from an uncontrolled study and therefore need to be interpreted with caution. A more recent study found that a large percentage of referrals, that would otherwise have been seen in secondary care, could adequately be dealt with in primary care by a practitioner with a specialist interest in allergy.^15^ A systematic review supports the need for alternative models of allergy care provision,^1^ however none of these models were tested with a control arm. Systematic reviews of disease‐specific nurse‐led clinics have shown high levels of patient satisfaction, however there remains a need for more robust studies.^16^, ^17^

Primary care based nurse‐led allergy clinics offer a new model of care for people with allergic conditions. This paper describes the feasibility of delivering and evaluating a nurse‐led allergy service to patients in Edinburgh, Scotland.

## 2 METHODS

### 2.1 Overview of methods

Our feasibility trial protocol is published elsewhere.^18^ We undertook a mixed‐methods study, through general practices in Edinburgh, Scotland between July 2017 and February 2021. General practices in South East and South West Edinburgh were invited to refer patients with specific suspected or clinician diagnosed allergic problems to a specialist nurse‐led allergy clinic (intervention) located in three hub practices (Phase 1). Referrals were paused in March 2020 due to COVID‐19 restrictions, and following an ethics amendment, were restarted in September 2020 using a secure remote model of care (Near Me – an NHS Scotland approved video consulting service) to the end of February 2021 (Phase 2).

### 2.2 The primary outcomes were

- Recruitment of practices to facilitate establishing the new service and make referrals.
- Referral and consultation rates for nurse‐led allergy clinic and retention rates.
- The change in disease‐specific quality of life questionnaires between baseline and 6–12 weeks post intervention.

### 2.3 Ethical considerations and permissions

We obtained written informed consent from patients and for young children from their parents. Children with capacity provided informed written consent and assent was obtained from their parents/carers. All participants were given a study ID and all data were anonymous to the research team.

### 2.4 Eligibility criteria

Patients were referred to the nurse‐led allergy clinic via secure NHS email. The eligibility criteria are summarised below.

#### 2.4.1 Inclusion criteria

- Children aged <36 months with suspected food allergy.
- Children aged <36 months with moderate‐to‐severe atopic eczema not responding to standard treatment.
- Children and young people up to 16 years of age with suspected allergic rhinitis symptoms not responsive to a combination of oral antihistamines and nasal steroids.
- Young people and adults (from 16 years of age) with a history of anaphylaxis or suspected anaphylaxis.
- Able to give informed consent/assent for children under 16 years.

#### 2.4.2 Exclusion criteria

- Children aged <36 months with suspected or confirmed non‐IgE‐mediated food allergy presenting primarily with gastrointestinal symptoms.
- Single urticarial reactions without obvious triggers.
- Non‐allergic chronic urticaria.
- Drug allergy.
- Well‐controlled allergic rhinitis, asthma or atopic eczema.
- Mild‐to‐moderate atopic eczema without any obvious allergic trigger.
- Localised insect sting reactions.
- Unable to give informed consent/assent.

### 2.5 Recruitment

Primary care‐based health care professionals (HCPs) were invited to complete a referral proforma for eligible patients via secure NHS email sent to the allergy nurse. If the referred patient met the inclusion criteria the patient/parent/carer were sent a patient information sheet and consent/assent form prior to being seen in the clinic. If they did not wish to take part in the study they were referred back to their HCP. Suitable patients were contacted by telephone or email and offered an appointment, where consent was taken, and baseline data were collected.

### 2.6 Intervention

The intervention is described in detail in the feasibility trial protocol.^18^ In summary, this was a nurse‐led allergy service in primary care. Nurses with a postgraduate allergy qualification and extensive secondary care experience were supported by a team of local specialist services, including paediatric and adult allergy, dermatology, ear, nose and throat (ENT) and respiratory medicine. Trial nurses took an allergy‐focussed history and clinical examination, with investigations as considered clinically appropriate (skin prick tests and blood samples) and provided a diagnosis, management advice and relevant education. A letter summarising the consultation was sent to the referring HCP including details of any medication(s) to prescribe. Patients were then discharged back to the care of their referring HCP. In some instances, further onward referral to secondary care services was required.

### 2.7 Recruitment to semi‐structured interviews

Patients, or parents/carers of young children, were invited to be interviewed and indicated on the consent form if they were happy to be approached by the qualitative researcher. If patients consented, their details were passed to the researcher who contacted them by telephone to arrange an interview at a time and place to suit them. Referring HCPs were asked by the allergy nurses if they would take part in an interview and, if so, their contact details were passed on to the qualitative researcher, who contacted them by telephone to arrange an interview.

### 2.8 Interviews

Patient/parent/carer participants were asked to give a description of their experience of their allergy in a semi‐structured interview conducted either face‐to‐face, by telephone or by email dependent on participant's choice. They were also asked about their clinic visit, and their views on current provision of allergy care. Interviews lasted between 15 and 30 min, and were audio‐recorded.

HCPs were asked about their experience of referring into the new clinic, how it impacted their workload, and their views on the provision of allergy care.

### 2.9 Data management and analysis

Disease‐specific quality of life (QoL) was measured at baseline and 6–12 weeks post intervention.^19^, ^20^, ^21^, ^22^, ^23^ At 6–12 weeks follow‐up, patients were asked to complete a satisfaction survey (adapted from Page et al 2008^24^), rating several aspects of the clinic with values from 1 (not happy) to 5 (very happy), either by telephone, email or face‐to‐face (via Near Me video consulting service from September 2020‐February 2021). No patient satisfaction surveys were sent to patients in Phase 2. Health care professional (HCP) satisfaction was measured via Online Survey towards the end of the referral window in Phase 1. Semi‐structured interviews with HCPs who referred into the specialist allergy clinic, and patients who attended the clinic were carried out at time points throughout the intervention. Primary outcome data were double entered in Microsoft (MS) Excel and errors corrected. Quantitative data was analysed using MS Excel. All the interview recordings were transcribed, checked, and anonymised. The interview transcripts were then analysed thematically, framed around both the research questions and themes arising from the data.

To examine socio‐economic inequality in the patient population Scottish Index of Multiple Deprivation (SIMD) 2020 was used, which was based on the 2020 census population.^25^ In Scotland in 2020 there were 6976 similarly sized areas of approximately 700 people, called data zones, where each had an allocated deprivation rank, the SIMD. SIMD rank for a data zone represents a comparative magnitude of deprivation across seven domains: income, employment, education, health, access to services, crime and housing. We used the SIMD ranks organised as quintiles (20% of the data zones), where quintile 1 represented the 20% of the data zones which were most deprived and quintile 5 the 20% data zones which were least deprived. SIMD quintile was obtained for each patient who attended the clinic by looking up their postcode of residence in the SIMD postcode lookup file.^26^ Edinburgh Southern had 96 data zones in 2020, of which one data zone was in the most deprived quintile. That most deprived data zone represented 1.04% of Edinburgh Southern and 0.07% of Scotland.^26^

## 3 RESULTS

### 3.1 Referrals

Of the 37 practices in the two referring localities, South East and South West Edinburgh, 35 referred patients to the allergy clinic. After assessing a total of 506 referred patients against the inclusion/exclusion criteria, 426 (84%) patients were allocated to receive the intervention in Phase 1 and 40/46 (87%) in Phase 2. The flow of patients from referral to follow‐up for Phase 1 and 2 are shown in Figure 1A,B, respectively.

FIGURE 1(A) Flow of patients through the trial in Phase 1. (B) Flow of patients through the trial in Phase 2

**Figure:**
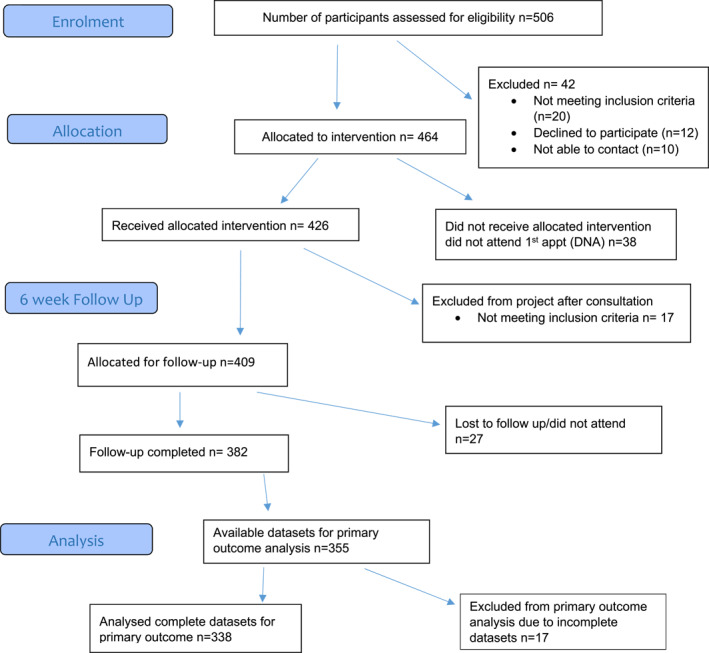

**Figure:**
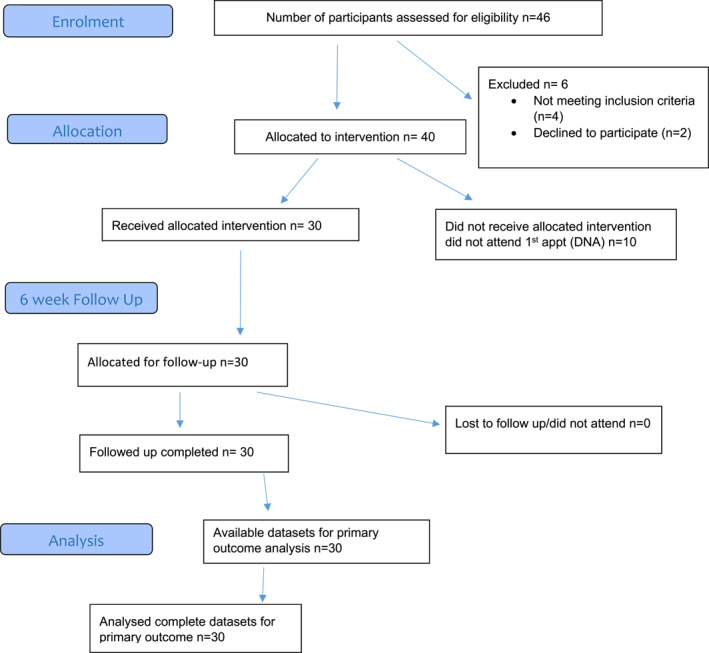

In Phase 1 the majority of eligible referrals were adults with a history of anaphylaxis or suspected anaphylaxis (*n* = 222/426, 52%), followed by infants under 36 months with suspected food allergy (*n* = 143/426, 34%), infants under 36 months with moderate‐to‐severe eczema (*n* = 51/426, 12%), and children and young people up to 16 years with allergic rhinitis (*n* = 10/426, 2%).

In Phase 2, 14 adult patients with a history of anaphylaxis or suspected anaphylaxis (*n* = 14/40, 35%) and 14 infants under 36 months with suspected food allergy (*n* = 14/40, 35%), were referred. Twelve infants under 36 months with moderate‐to‐severe eczema (*n* = 12/40, 30%) were referred and there were no referrals for allergic rhinitis.

In Phase 1, GPs referred most patients to the clinic (*n* = 320, 69%), followed by health visitors (*n* = 129, 28%) and practice or community nurses (*n* = 17, 3%). In Phase 2, both GPs and health visitors referred 20 patients.

### 3.2 Demographics of referred patients

In both phases of the study, similar numbers of males and females were referred, with similar percentages of adults and children in Phase 1, but more children under 16 years (65% than adults in Phase 2 [Tables 1 and 2]).

**TABLE 1:** Age and sex of patients seen in Phase 1

Age	Total	Female	Male
Adult age 16 years and over	221	152	69
Child up to 16 years	205	88	117
Total patients	426	240 (56%)	186 (44%)

**TABLE 2:** Age and sex of patients seen in Phase 2

Age	Total	Female	Male
Adult age 16 years and over	14	10	4
Child up to 16 years	26	8	18
Total patients	40	18 (45%)	22 (55%)

**FIGURE 2:**
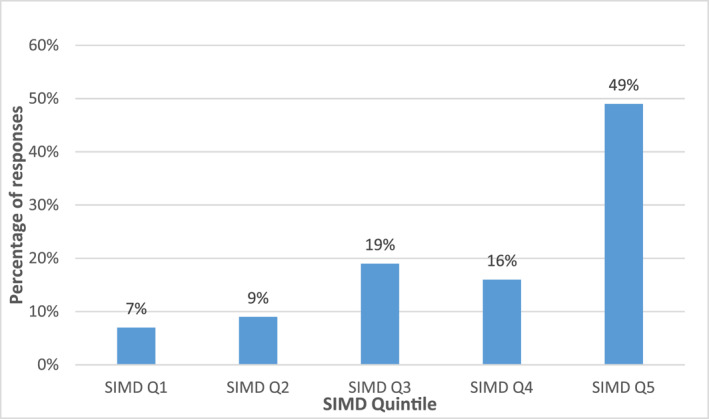
Percentage of patients seen by Scottish Index of Multiple Deprivation quintile

The majority (49% in Phase 1% and 53% in Phase 2) of patients referred to the clinic were in SIMD quintile 5, representing the 20% least deprived areas in Scotland (Figure 2).

### 3.3 Quality of life

Complete data were available for 338/426 (79%) of patients seen at baseline and follow‐up in Phase 1 and 30 (75%) in Phase 2. The QoL scores at follow‐up were reduced or unchanged in all measures, with the exception of the teenage food allergy QoL tool in Phase 1 (Tables 3 and 4). This indicates improved or unchanged QoL for almost all measures.

**TABLE 3:** Phase 1 ‐ Disease‐specific quality of life score at baseline and 6–12 weeks follow‐up

	Baseline	Follow‐up
Food allergy QLQ^a^ adult form (scale 1–7^c^) (*n* = 167)	4.1	3.3
Food allergy QLQ^a^ parent form (scale 0–6^c^) (*n* = 115)	1.8	0.7
Food allergy QLQ^a^ teenage form (scale 1–7^c^) (*n* = 5)	4.3	4.6
Mini RQLQ^b^ (scale 0–6^c^) (*n* = 7)	3.0	1.3
Dermatitis score (scale 0–4^c^) (*n* = 44)	2.0	1.3
Life quality score (scale 0–30^c^)	9.6	4.6

^a^ QLQ, quality of life questionnaire.

^b^ Rhinoconjunctivitis quality.

^c^ High QLQ scores indicate low QOL.

**TABLE 4:** Phase 2 ‐ disease‐specific quality of life score at baseline and 6–12 weeks follow‐up

	Baseline	Follow‐up
Food allergy QLQ^a^ adult form (scale 1–7^b^) (*n* = 11)	4.7	3.9
Food allergy QLQ^a^ parent form (scale 0–6^b^) (*n* = 11)	1.6	1.2
Food allergy QLQ^a^ teenage form (scale 1–7^b^) (*n* = 1)	5.5	1.9
Dermatitis score (scale 0–4^b^) (*n* = 7)	2.4	1.7
Life quality score (scale 0–30^b^)	11.9	6.6

^a^ QLQ, quality of life questionnaire.

^b^ High QLQ scores indicate low QOL.

### 3.4 Secondary outcomes

#### 3.4.1 Satisfaction

##### Patient satisfaction survey

Immediately after their 6–12 weeks follow‐up appointment, 371 patients/parents/carers completed a satisfaction (Table 5). Between 64% and 91% of participants were very happy^5^ with the individual aspects, with the highest scoring domain being the general information given by the nurse.

**TABLE 5:** Patient satisfaction with aspects of the allergy clinic measured in percentages

	5 (very happy)	4	3	2	1 (not happy)	Missing response
	(%)					
Length of time from last seeing your GP to being seen in the clinic	76.5	13.4	4.0	0.8	0	5.1
Examination	74.4	9.1	1.0	0	0	15.4
Discussion with you of treatment options	87.3	8.1	1.3	0	0	3.2
Discussion with you of how to use the treatments	89.2	7.0	1.1	0.3	0	2.4
General information given by the nurse	90.8	6.7	0.8	0	0	1.6
Ease of getting your prescription	64.9	7.3	5.7	1.1	0.5	20.5
How well the treatment worked	63.6	16.2	4.0	0.5	0	15.6

When asked about self‐assessed improvement of their allergy, 42% (*n* = 155) of patients said their allergy was much better since attending the allergy nurse clinic, 40% (*n* = 148) said they were slightly better, and 16% (*n* = 59) said there was no change. Less than 2% said their allergy was worse or slightly worse. The majority of patients (92%) said there were able to keep to their personal management/treatment plan advised by the allergy nurse.

##### HCP satisfaction survey

Twenty one HCPs who had made referrals into the service completed a satisfaction survey (12GPs, 6 Health visitors, 1 practice nurse, 1 community nursery nurse, 1 other). The majority of HCPs had referred between 1 and 10 patients into the clinic, two had referred 11–20 and 2 had referred 21–30 patients. Most respondents found the referral process very easy, but requested as the main improvement to set up the clinic on the Scottish Care Information (SCI) Gateway, a national system that integrates primary and secondary care systems using highly secure Internet technology.

In a satisfaction survey, HCPs scored all aspects of their patients care in the nurse‐led allergy clinics very highly with mean scores between 4.3 and 4.7 (scale 1–5, not happy ‐ very happy) (Table 6).

**TABLE 6:** HCP satisfaction survey

Category of care	Mean score
Referral time	4.6
Examination/assessment of your patient's condition	4.7
Appropriateness of treatments of recommended by nurse	4.7
Documentation of how to use the treatments	4.7
Ease for patient to get prescription	4.7
Cost effectiveness of the treatment regimens	4.3
Clinical effectiveness of the treatment regimens	4.7

*Note*: (1 = not happy, 5 = very happy).

When asked about the best aspect of the clinic, HCP free text responses focussed on ease of access to the clinic, quick appointment times and reduced waiting times, as well as seeing an experienced HCP and having time for discussion (See Box 1 for full list).

When asked about the worst aspects of the clinic, the response were limited to requests for the use of SCI‐Gateway for referrals, discharge summaries including prescribing information, and for shorter waiting times for clinic appointments.

**Box 1: HCP free text responses to satisfaction survey**

♦ Ease of access, quick appointment turnaround and reduced waiting time.
♦ being seen quickly by knowledgeable professionals and having more time.
♦ parents appreciate the ability to spend time discussing food exclusion/reintroduction.
♦ much needed allergy service that is otherwise lacking.
♦ access to an experienced individual who sees patients in a timely fashion.
♦ feedback by email so you know the findings and plan.
♦ The short waiting times for parents. Also the clear information provided as some parents have previously been left without any support. When they have visited the allergy nurse led clinic they are given clear information and guidelines regarding their child allergy.
♦ thorough assessment of patients and useful advice for prescribing.
♦ prompt access for a difficult problem.
♦ useful tool for patients with anaphylaxis. This is a big gap in referral pathways otherwise.

### 3.5 Patient and family carer interviews

Sixteen patients and/or family carers were interviewed with a range of allergic disease as shown in Box 2.

All the participants interviewed were impressed by the new clinic and very pleased with their experience there. They said that being able to spend time with an experienced specialist nurse led to improvements in their knowledge about their allergies, a greater feeling of control over them, and a consequent improvement in their QoL. Participants liked the fact that the consultation focused not just on their physical symptoms, but also on practical ways to minimise their impact on their daily lives, and on ways to cope with the anxieties raised by these symptoms. All said that they felt more supported, and appreciated the convenience of a local clinic.

**Box 2: Description of patients interviewed by allergic condition and sex**

4 adult males with allergies/anaphylaxis;

4 adult females with allergies/anaphylaxis;

1 father of a toddler with food allergies and eczema.

5 mothers of a baby/toddler with food allergies.

1 teenage boy (interviewed with his mother) with anaphylaxis.

1 teenage girl with food allergies.

**Box 3: Themes arising from patient interviews**

**Table:** 

Theme	Sub‐theme
Organisation of clinic	Ease of access to the clinic short waiting times
Consultation	Knowledge of nurse length of consultation tests, results available during consultation
Patient empowerment	Improved knowledge and control reassurance

#### 3.5.1 Organisation of the clinic

Participants commented on two particular aspects of the organisation of the clinic: its geographical location and the speed with which they were seen. People found it easy to attend the clinic because it was situated in their local area, thus eliminating transport problems, and because it was based in a familiar location, either their own, or a nearby, GP practice. Some people specifically remarked that they might not have attended had the clinic been hospital‐based.

Having it local was incredible. It was so convenient. (Participant 2)

I guess I might have gone somewhere further away, but it’s very handy given that it’s so close to home. (Participant 3)

It was a short bus ride…if it had been across town I might have thought it was a bit far to travel. (Participant 5)

I would probably still have gone, even if it was at one of the big hospitals, but I wouldn’t have felt so great about it… I just literally live round the corner from the GP. (Participant 4)

In addition, participants were impressed by the short time they had to wait between being referred by the GP and seeing the specialist nurse. Those who had attended specialist clinics elsewhere were particularly pleased about the difference with this clinic. People spoke of being very anxious about their allergies, and the impact on their daily lives (eating, sleeping, interacting with others) and so had found it difficult in the past having to wait a long time before being able to speak to a specialist for advice.

The clinic was really nice and having it very close, obviously the quick turnaround of just a week or two was amazing. We used to have to wait months for an appointment and stuff back home so it was really, really good. (Participant 6)

The GP referred me and I had the first meeting within 10 days, which was brilliant. (Participant 14)

#### 3.5.2 The consultation

All the participants found the consultation helpful. The helpful aspects that people highlighted were: the amount of time they were able to spend with the nurse and their specialist knowledge of allergies and daily living with an allergic condition. Participants also appreciated having their tests done in the clinic, and some results being immediate.

It was a very positive experience… because it was very handy to actually do the test, which is more than just a blood test, and get that much information … I don’t think I had enough information before. (Participant 3)

I would say that it (the consultation) was a great experience. It was really helpful to reassure me that I am still extremely allergic to certain things. (Participant 4)

It was very helpful. I think that was part of the issue, that you just want a bit of advice. (Participant 12)

#### 3.5.3 Length of the consultation

The participants commented that the consultation lasted longer than they had expected, and that they therefore had time to discuss all the aspects they were concerned about. Many contrasted this with their previous, and often repeated, visits to see a GP where they felt rushed, and where their concerns had not really been attended to as they had hoped. Several said that they felt as if they were bothering the GP with concerns that were perhaps minor to him/her, but important to them, and came away from GP consultations still feeling worried and anxious.

The nurse was like really, really helpful and she was really sweet as well. She made me feel really welcome, and she wasn’t in a hurry, which I think is a really important thing. (Participant 4)

it's not similar, again, to a GP where you’re in and out in five minutes. There, it kind of felt like they took the time that was needed, there was no rushing, and that was nice. (Participant 2)

The fact that, I think, we only had a 15–20 minute appointment scheduled, but in fact it took an hour was just, me with lots and lots of questions, and her, just answering all these questions and giving more…way more info than I could’ve imagined.” (Participant 7)

In addition, because the nurse had such specialist knowledge, participants felt they had received a consultation personal to them and their experience, and gained much additional knowledge and reassurance. They felt it was very important that the nurse showed a genuine understanding of daily life with allergies, and took their concerns seriously.

I have never in all these years, I've never spoken to anybody who's had such a complete and comprehensive understanding of allergies and how they interconnect. And that for me was a revelation. (Parent of Participant 8)

The most beneficial thing about the clinic was having the time to talk it through properly with someone who understands it. (Participant 14)

When asked if they would prefer the clinic to be run by a doctor, all the participants were happy and confident in the nurse.

I was quite happy to see a nurse. I got the feeling she knew what she was talking about. (Participant 5)

I wouldn’t mind who was running the clinic. The nurse was very knowledgeable and could answer all my questions. (Participant 14)

The GP and the health visitor can only do so much, while the nurse is an expert. I felt like I got better care from her. I trust her. (Participant 13)

#### 3.5.4 Patient empowerment

When participants talked in general about the impact of their visit to the clinic, the aspects they found most helpful were two‐fold: practical and emotional. Practically, participants valued the increased knowledge and understanding of their allergies, and the information on how to cope with them, that they gained from the consultation.

I found the allergy clinic very useful. They were able to inform me a lot more about what to do with these reactions. (Participant 2)

I don’t think I had enough information before to really know what my condition is and what it’s about. (Participant 3)

Participants were pleased to have been given advice on how to cope with their particular allergies and social situations, and felt they now had a clear plan of what to do on a day to day basis. Many found being given training in how to use an adrenaline auto‐njector very reassuring, and also felt more in control once they had practical tips and ideas on how to deal with the issues worrying them.

Also some practical things. She gave me training on the Epipen, because I’ve never had to use it… and also She was saying things like it’s better if you do it when you’re sitting down, because you might collapse….., and I’d never thought of that. (Participant 3)

a lot of tips to keep it more under control, and I have been able to sleep a lot better and I’m not itchy, I don’t sneeze as much, at all. (Participant 6)

I was prescribed daily antihistamines, which have really helped, I think, and a nasal spray, so I was given more stuff to cope with the allergies I already had, informed more about them, and it was just nice to have someone take that time to help me with this thing that plays a big part in my life rather than, yeah, just eat something and if it's bad, don’t eat it again. (Participant 2)

I was very anxious and afraid that I would aggravate his skin (child). I feel I have a plan now, and someone I can call if his allergies get worse. (Participant 13)

Participants felt that, as a result of having gained more knowledge and understanding of their allergies, they felt more in control of them, and so were more relaxed and confident in their everyday lives. They spoke about having a plan and feeling less anxious, feeling that they were in control of their symptoms in their daily lives, rather than the other way round, which was their previous experience.

I feel more confident now, I guess, because I now have a broader picture of what I'm actually allergic to… I feel like I've got a clearer picture. (Participant 2)

(the clinic) was extremely useful because I actually started to understand things more… so I feel like I’m more in control now, because I know what is happening and what I should avoid. (Participant 3)

The nurse gave us a plan for how to deal with the allergy… I got the feeling she knew what she was talking about… and that helped to reassure me. (Participant 5)

(the nurse) was great. I found out my child’s allergies are not as bad as I thought. She put my mind at ease. I’d been too scared to introduce any new foods but with her help he is now eating a more balanced die.t (Participant 13)

### 3.6 HCP interviews

Nine HCPs were interviewed, five referring HCPs (2 HV, 3 GPs), one practice manager and the two specialist allergy nurses, one of whom took part in two interviews, one in the middle of the study and one at the end.

All the HCPs interviewed were very positive about the service. They felt that the clinics addressed an existing gap in the services for people with allergies, who were previously having to wait a long time to see a specialist, and were often left feeling anxious and uncertain what to do in the meantime.

I think it’s a good addition to our service here. (GP2)

I feel it’s a really helpful resource. Many adults with a history of allergies had no service to go to prior to this clinic. (GP2)

In my specific caseload I’ve found the clinic really useful… and the clients also feed back that it’s been a great support. (HV2)

It took me a bit of a while to work out what it actually was but as soon as I got my head around that I thought that’s excellent, and it has worked wonders for children and parents on my caseload. (HV1)

#### 3.6.1 The process

All the professionals found the process of referring to the clinic clear and straightforward, and were easily able to communicate with the clinic nurses, and pick up the care of their patients again once they were discharged.

I found the referral process very straightforward, and the proforma quite clear…it has been quite a smooth process. (GP2)

The nurse dictates a very clear letter after the clinic with recommendations and if a prescription is needed so the patients are easy to manage again on discharge from the clinic. (GP 2)

I would say that the referral form itself is quite simple. (GP1)

They also reported that they and their patients were happy to have the clinics being nurse‐led, because the nurses had specialist knowledge.

I like it being a nurse‐led service. (HV2)

They are quite happy to see the nurse, because she is very knowledgeable. (HV2)

#### 3.6.2 The clinic

Whilst the clinic may not have reduced workload for HCPs, it filled a gap for the young adults, as there were no transition or adult allergy services available in Edinburgh, as well as being local and based in a hub general practice making it more accessible.

I think it will have reduced my workload. (HV2)

I would say its impact is neutral …as although we see a lot of patients with allergies there are relatively few in the grand scheme of things that need a formal allergy review…. The majority can probably just be managed pragmatically. (GP1)

It fills a gap in the service. As I said a lot of these young adults previously had nowhere to go for a history of anaphylaxis or for young children who start with eating issues, so I think it’s a great place for them. (GP2)

It feels more accessible than what we had before… for some clients that’s on a practical level of just trying to get to where the clinic is… but it also just feels more accessible as a service. (HV2)

In terms of improvements to the service, HPCs felt that although the referral system was straightforward and easy to use, it would be improved by implementing the more recognised referral pathway via SCI Gateway.

If it could be continued, then it could be put on SCI‐Gateway in this clinic and that will be even better. (GP2)

In terms of improvements I would say having a formalised referral on SCI Gateway would help. (GP1)

It would be helpful to have an immediate outpatient letter, so that the nurse could just write her recommendations and prescription and give it to the patient … this could help expedite a lot of prescriptions as well. (GP2)

## 4 DISCUSSION

This large feasibility trial has demonstrated that it is possible to recruit patients, deliver the intervention and retain individuals in a nurse‐led primary‐care based allergy clinic. Our data indicate that the model of care developed was acceptable to both patients/carers and HCPs, and that this has the potential to result in improvements in disease‐specific QoL.

There are a number of strengths to our work. We developed the particular model of care deployed after careful consideration of a range of ways of providing this service. Our eligibility criteria were based on an in‐depth appreciation of some of the key challenges patients/carers describe with provision of allergy care in primary care settings and the clinical priority areas identified by primary care clinicians. The large numbers of patients seen, the use of disease‐specific QoL measures, and the opportunity for in‐depth assessment of perspectives derived from qualitative interviews are additional major strengths. Finally, we demonstrated an ability to move the clinic to a remote model of care, which was important in the pandemic era.

There are some limitations to our work that need to be considered. Chief amongst these is that we did not get complete follow‐up data on all participants. This is important, because participants who did not complete the post‐intervention follow‐up assessments may have been less likely to have valued the intervention and/or improved, potentially introducing bias. It is also important to note that although we had four clinical conditions that we sought to improve, over 80% of referrals were for suspected food allergy and anaphylaxis. Numbers of participants recruited with severe childhood eczema or seasonal allergic rhinitis unresponsive to pharmacotherapy were therefore small.

Moving forward, these findings provide a firm basis to move to a pilot randomised controlled trial followed by a possible definitive randomised controlled trial. Given the referral and recruitment patterns seen, it would seem prudent to focus follow‐on work on food allergy and anaphylaxis; this would also mean the need to work with fewer disease‐specific QoL measures. Although retention was good overall, there is a need to improve this, which is probably best achieved by emphasising the importance of these final study outcome measures at the time of recruitment and also by making it possible to complete questionnaires through a range of channels, including in person, over the phone or over the Internet through a smart phone, tablet or personal computer. Future work also needs to involve a formal health economic evaluation from the perspectives of the NHS.

## 5 CONCLUSION

We have demonstrated the acceptability, feasibility and potential effectiveness of a nurse‐led primary care‐based allergy clinic for patients with suspected/confirmed food allergy, eczema, allergic rhinitis and anaphylaxis. There is now a need to build on this work to formally evaluate the effectiveness of the intervention through a pilot and then definitive randomised controlled trial.

## AUTHOR CONTRIBUTIONS

Aziz Sheikh and Jurgen Schwarze conceived and led this study and together with Lynn Morrice secured funding for this project. Vicky Hammersley was the project manager. Margaret Kelman and Susan Harley were the trial nurses. Mome Mukerjhee supported quantitative data analysis. Marilyn Kendall undertook the interviews and led the qualitative evaluation. All authors contributed to the writing of the manuscript.

## CONFLICT OF INTEREST

The authors declare that there is no conflict of interest.

## Supporting information

Supporting Information S1
