# Supplementary material for: Mixed‐methods evaluation of a nurse‐led allergy clinic model in primary care: Feasibility trial
Source: Clin Transl Allergy. 2022 Aug 9;12(8):e12180. doi: 10.1002/clt2.12180 (PMC9362986; doi:10.1002/clt2.12180)
Supplement: Supplementary file 1 — Supporting Information S1 [file CLT2-12-e12180-s001.docx]

Patient details

Name male / female

Address

Date of birth Age

Phone number email

Reason for referral Please Tick

- Infant under two with food allergy
- Infant under two with moderate to severe eczema not responding to treatment
- A child or young person (under 16) with rhinitis symptoms not responding to a

combination of oral antihistamines and nasal steroids

- A young person/ adult (16+) with a history of anaphylaxis or suspected anaphylaxis

Would you have referred this patient to secondary care if you did not have access to this clinic?

Yes / no / no secondary service to refer patient to / other (please circle)

If answered yes please state which speciality …………………………………………………

**Please note this question must be answered for all referrals to the allergy clinic**

Relevant past medical history and any recent investigations

Current medication

Findings on examination and any known allergies

Other supporting information

Gp Signature

Date

Practice stamp

**Criteria checklist - for clinic referral**

Referral guidance for referring into nurse led allergy clinic

**Referring to Allergy Clinic**

**Does the patient fall in to one of the categories listed below ?**

- **Infant under two with suspected food allergy**
- **Infant under two with moderate to severe eczema not responding**

**to treatment**

- **A child or young person (under 16) with rhinitis symptoms**

**unresponsive to a combination of oral antihistamines and**

**nasal steroids**

- **Young person or adult (16+) with a history of anaphylaxis**

**or suspected anaphylaxis as per definition of anaphylaxis below**

Anaphylaxis is likely when the following two criteria are met:

Sudden onset and rapid progression of symptoms Life-threatening airway

and/or breathing and/or circulation problems

The following supports the diagnosis:

- Additional skin and/or mucosal changes (flushing, urticaria, angioedema)
- Gastrointestinal symptoms (vomiting, diarrhoea, abdominal pain)
- Exposure of a person with allergy to their known allergen (CYANS 2012)

Referral checklist adapted from Levy et al 2009(1)

1. Levy ML, Walker S, Woods A, Sheikh A. Service evaluation of a UK primary care-based allergy clinic: quality improvement report. Primary Care Respiratory Journal. 2009;18:313.
